# Internalized Homonegativity, Emotion Dysregulation, and Isolating Behaviors Perpetration among Gay and Lesbian Couples

**DOI:** 10.3390/ijerph20021593

**Published:** 2023-01-16

**Authors:** Tommaso Trombetta, Virginia Balocco, Fabrizio Santoniccolo, Maria Noemi Paradiso, Luca Rollè

**Affiliations:** Department of Psychology, University of Turin, via Verdi 10, 10124 Turin, Italy

**Keywords:** internalized homonegativity, emotion dysregulation, intimate partner violence, isolating behaviors, controlling behaviors, same-sex intimate partner violence, sexual minorities

## Abstract

Same-sex intimate partner violence (SSIPV) is a serious health concern and may manifest in various forms. Nevertheless, controlling behaviors of isolation are still poorly investigated. Due to their marginalized status, sexual minorities can face SSIPV-specific risk factors, such as internalized homonegativity, as well as general psychological stress factors, such as emotion dysregulation. While the literature is growing, there is still a lack of understanding of the complex pathways linking sexual minorities and minority stress to IPV and isolating controlling behavior. To fill this gap, we explored the relation between internalized homonegativity and controlling behaviors of isolation, assessing the mediating role of emotion dysregulation. In total, 120 gay and lesbian people (mean age = 33.8, SD = 11.5) involved in a same-sex relationship participated in the study. Results showed a direct and positive association between internalized homonegativity and difficulties in emotion regulation and a direct and positive association between emotion dysregulation and controlling behaviors of isolation; the mediating role of emotion dysregulation in the relation between internalized homonegativity and isolating controlling behaviors was supported as well. Emerging results can provide valuable information at the clinical level, although further studies are needed to confirm these preliminary findings.

## 1. Introduction

Intimate partner violence (IPV) is a serious public health concern affecting sexual minorities to the same extent as heterosexual people, if not even more [1,2,3]. Only in the past few decades researchers turned their attention to same-sex intimate partner violence (SSIPV; [4]). Nevertheless, IPV among same-sex couples remains poorly discussed on a public level and bias in media representations have been documented, which contribute to the poor understanding of the phenomena [5,6]. The lack of research on SSIPV may also have perpetuated the marginalization of sexual minorities resulting in a shortage of service provision, public agendas, and funding, especially when compared to the response dedicated to heterosexual IPV [1]. Moreover, even if theories developed by research with heterosexual couples can often be applicable to LGB couples, they fail to represent sexual minorities’ unique experience [7]. Intervention and prevention programs for IPV in same-sex couples frequently refer to data collected on the heterosexual population. Due to this heteronormative bias, prevention and intervention programs are often not effective in capturing the needs of sexual minority people [8]. Abusive dynamics within same-sex couples can be fueled by the heterosexist context and internalized stigma [9]. When analyzing SSIPV, some specific violent tactics may be involved, such as threatening to disclose a partner’s sexual identity (outing), undermining a partner’s identity as an LGBTQ person [10], or isolating the partner from specific sources of support such as the LGBTQ communities [9,11]. These modalities are closely linked to the minority status and marginalized conditions of the victims which can be used by the abuser to gain control or elicit feelings of shame [12,13]. Sexual minorities also face several help seeking barriers making them less likely to seek help [14,15]. Stereotypes and discrimination against the LGBTQ community can spread not only on a social level but also among mental health, legal and medical professionals [16,17]. As a result, members of sexual minorities who experience violence may fall back on informal sources of help and avoid services for fear of encountering a hostile, unprepared, and retraumatizing environment [15,18]. 

Beyond similarities between IPV in sexual minorities and heterosexual people, due to their marginalized status, sexual minorities can also face SSIPV-specific risk factors such as minority stress [19]. Minority stress refers to an additional stress factor, given by the minority status, that is chronical and deeply rooted in the heterosexist context [20,21]. Among the dimensions comprised in the minority stress model (i.e., experiences of discrimination, perceived stigma, internalized homonegativity, sexual identity concealment), internalized homonegativity can influence IPV perpetration in sexual minorities [22,23,24,25]. Internalized homonegativity refers to the negative affect and attitudes directed toward the self and one’s personal sexual orientation [20,26]. Several studies identified a positive association between higher levels of internalized homonegativity and SSIPV perpetration [24,27,28,29,30,31]. However, the mechanisms by which this happens are not fully understood yet. 

A recent contribution to the minority stress theory comes from Hatzenbuehler [32]. The author proposed the Psychological Mediation Framework (PMF) with the aim of expanding Meyer’s original model [20,26] and taking into consideration both group-specific stressors, general psychological processes, and their interdependence. The PMF gives focus not only on unique stress factors that sexual minorities may encounter, but also on more general risk factors that may represent psychological and social challenges. Specifically, the model suggests that stigma-related stressors make sexual minorities more vulnerable to general risk factors such as emotion dysregulation [32]. The PMF has until now been applied to the study of LGBTQ health inequalities, by postulating that both general stressors and minority specific factors contribute to health inequality in this population and that the effect of minority stress on well-being is mediated by psychosocial variables such as feelings of hopelessness, low self-esteem, social isolation and emotion dysregulation [33,34,35,36,37,38]. So far, a body of research has focused particularly on emotion dysregulation, which has been reported to mediate the relationship between minority stress, and in particular internalized homonegativity, and psychological distress [37,39,40,41,42]. Minority stress, conceived as a chronic stressor that contributes to ego depletion [43], can undermine functional emotion regulation strategies, promoting maladaptive emotion regulation strategies used to relieve the conflict between one’s perceived sexual orientation and discrimination [44,45]. Emotion dysregulation has, in turn, been associated with higher levels of psychological distress and depression [39,46,47]. In addition, emotion dysregulation has been found to be related to IPV perpetration in several studies [48,49]. However, as stated by the World Health Organization [50], IPV refers not only to acts of physical, psychological, and sexual violence but also to controlling behaviors, including isolation, stalking, and restricting access to health care, education, employment, or financial resources. Among these, controlling behaviors of isolation are still poorly investigated [51,52] and different kinds of intimate partner violence are frequently mixed together by scholars [53]. While controlling behaviors of isolation certainly take place within heterosexual relationships too, they can be particularly detrimental and underreported when involved people are members of a marginalized minority [13,17,54,55]. Sexual minorities already face heightened risk of experiencing rejection from their close backgrounds and families of origin [9,56], of living alone without immediate family system in midlife and older age [57], and are more likely to report low levels of social support in both their peer and family contexts [58,59]. Controlling behaviors of isolation are especially concerning given their deleterious effect of increasing victim’s dependence on the abuser and thus heightening the risk of further violence exposure [1]. 

In addition, to what we know, except for one recent study on IPV victimization among sexual minority men, which highlighted associations between childhood sexual abuse, intimate partner violence victimization, internalized homophobia and emotion regulation difficulties [60], no studies have assessed the application of the PMF to SSIPV perpetration. In particular, the mediating role of emotion dysregulation in the association between internalized homonegativity and controlling violence has not been tested yet.

To address this research gap, the present study aims to evaluate the application of the PMF to the SSIPV context, with a particular focus on controlling behaviors of isolation. We therefore tested whether emotion dysregulation can be a significant mediator in the relationship between internalized homonegativity and SSIPV. Emerging results can provide valuable information at a clinical level, with the aim to tackle SSIPV perpetration and reduce relapses as well as increase individual and relational well-being among sexual minority people.

### Hypotheses

**Figure 1:**
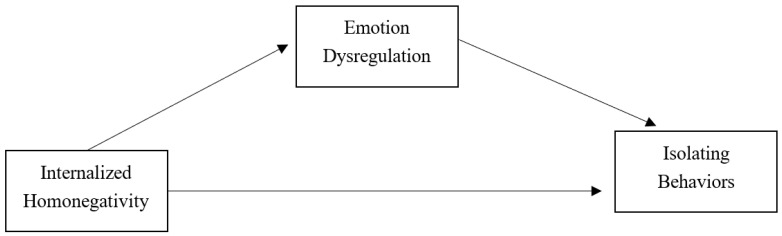
Hypothesized model. Note: mediation model with emotion dysregulation as mediator and sex, sexual orientation, education level, and economic condition as controlling variables.

The hypotheses for the present study are the following and are presented in Figure 1:

**Hypothesis** **1** **(H1):** *Internalized homonegativity is expected to be directly and positively associated to isolating behaviors perpetration*.

**Hypothesis** **2** **(H2):** *Internalized homonegativity is expected to be directly and positively associated to emotion dysregulation*.

**Hypothesis** **3** **(H3):** *Emotion dysregulation is expected to be directly and positively associated to isolating behaviors perpetration*.

**Hypothesis** **4** **(H4):** *Emotion dysregulation is expected to mediate the association between internalized homonegativity and isolating behaviors perpetration*.

## 2. Materials and Methods

### 2.1. Participants

A total of 141 participants completed the questionnaire. Twenty-one participants were not involved in a same-sex couple relationship and were excluded. The final sample included 120 participants involved in a same-sex couple relationship (62% males) between the ages of 20 and 76 (Mean age = 33.8, SD = 11.5). The respondents′ sociodemographic characteristics are presented in Table 1.

### 2.2. Procedure

Study procedures are in accordance with the ethical standards of the APA and the 1964 Declaration of Helsinki. The questionnaire was prepared by the research team, using validated scales whenever possible, and translated into Italian. Data were collected from July 2021 to April 2022 through an online survey conducted on LimeSurvey. The questionnaire contained general information about the study and an invitation to participate, which was distributed by the research team members to their personal, professional, and social networks through email and word of mouth. Participation was voluntary and anonymous. Before beginning the questionnaire, participants received an informed consent form describing the aims of the study and the content of the survey, risks, benefits, privacy, names of research institutions, and contact information for the study team head. Completion of the questionnaire took approximately 15 min. The study was approved by the Bioethical Committee of the University of Turin.

### 2.3. Instruments

Internalized Homonegativity: The Internalized Sexual Stigma for Lesbian and Gay Men (MISS-LG; [61]) was used to assess internalized homonegativity in our study. Each item is rated using a 5-point Likert-type scale, ranging from “Totally disagree” to “Totally agree”. In accordance with Lingiardi and colleagues’ specifications [61], we obtained the total score by adding all the items. In our sample, Cronbach’s alpha coefficient of the total score was 0.72.

Emotion Dysregulation: The Difficulties in Emotion Regulation Scale (DERS; [62]) in its Italian version [63,64] was used to assess emotion dysregulation. The DERS is composed of six subscales: non-acceptance of emotional responses (Nonacceptance), difficulties engaging in goal-directed behavior, impulse control difficulties (Impulse), lack of emotional awareness (Awareness), limited access to emotion regulation strategies (Strategies), and lack of emotional clarity (Clarity). Participants rated each item on a five-point Likert scale ranging from 1 (“Almost never”) to 5 (“Almost always”). For the current study, the total score was considered, and Cronbach’s alpha was excellent (0.94).

Controlling Behavior Perpetration: To assess controlling behavior perpetration, the Controlling Behavior Scale (CBS-R; [65,66]) was used. It consists of five subscales (economic, threats, intimidation, emotional, and isolation), which participants responded to considering both victimization and perpetration. For the current study, only perpetrated isolating behaviors were considered. Participants rated each item on a five-point Likert scale ranging from 0 (“Never”) to 4 (“Always”). The Cronbach’s alpha was good (0.71).

The items in each scale were all treated as continuous variables and were summed to obtain a total mean score.

The following sociodemographic variables were included as control variables according with the literature on IPV perpetration: sex, educational level, economic condition. 

### 2.4. Data Analysis

Statistical analyses were performed using the Statistical Package for the Social Sciences (SPSS 28.0) and a mediation analysis was tested using Hayes’ [67] PROCESS (Version 4.1, Model 4) to test direct and indirect effects. Frequencies, means, and standard deviations were calculated to summarize the variables included in this study. Pearson′s correlation (r) was used to test the relationship between variables, and results were interpreted according to Cohen’s [68] conventions. T-tests were used to assess gender differences in controlling behaviors perpetration. The reliability of each scale was determined using the Cronbach’s alpha coefficient. 

Sex, sexual orientation, educational level, and economic condition were used in the model as control variables.

As recommended by Tabachnick and Fidell [69], the studied variables were tested for the assumptions of normality and multicollinearity. Since the data violated the multinormality condition we used a robust estimator to test the significance of the model. To assess the mediation model according to our hypotheses we used bootstrap estimation to test the significance of the indirect effects [67] with 5000 samples, and we computed the bias-corrected 95% confidence interval (CI) by determining the effects at the 2.5th and 97.5th percentiles; when 0 was not included in the CI, the indirect effects were significant.

## 3. Results

Frequencies and mean scores of scale study variables are reported in Table 2. No significant differences emerged between female and male participants in isolating behaviors perpetration. 

Table 3 reports bivariate correlations among scale scores. The results showed a positive correlation between internalized homonegativity and emotion dysregulation (r: 0.46, *p* < 0.01) and between emotion dysregulation and isolating behavior perpetration (r: 0.23, *p* < 0.05).

To test our hypotheses, we conducted a mediation model. Internalized homonegativity was the independent variable, emotion dysregulation the mediators and isolating behavior perpetration the dependent variable.

After we checked for control variables and according to the literature on this field, we included in the final model: sex, sexual orientation, educational level, and economic condition as covariates.

The hypotheses in the current study were partially confirmed. In contrast to H1, no significant direct association emerged between internalized homonegativity and isolating behavior perpetration (b: 0.06; se: 0.45; *p*: 0.564). Instead, a direct and positive association emerged between internalized homonegativity and emotion dysregulation (b: 0.44; se: 0.07; *p* < 0.001) in accordance with H2, and a direct and positive association emerged between emotion dysregulation and isolating behavior perpetration (b: 0.21; se: 0.42; *p* < 0.05) in accordance with H3. There were no significant associations between the sociodemographic variables (sex, sexual orientation, educational level, and economic conditions) and emotion dysregulation or isolating behavior perpetration. The model explained 6.4% of the variance for isolating behavior perpetration (F(6, 113) = 17.72; *p* < 0.001).

The indirect effect was also significant in accordance with H4. In detail, a positive indirect effect emerged between internalized homonegativity and isolating behavior perpetration through the mediation of emotion dysregulation (b: 0.10; Bootstrap se: 0.05; CI: 0.006; 0.19). Finally, neither direct (b: 0.68; se: 0.47; *p* 0.146) nor total (b: 0.26; se: 0.45; *p* 0.563) effects of internalized homonegativity and controlling behavior perpetration were significant. 

## 4. Discussion

The current study assessed the application of the PMF [32] to SSIPV, focusing on controlling behaviors of isolation perpetration, which are still poorly investigated and can be particularly detrimental to members of a marginalized minority [17,54]. For this purpose, we explored the role of internalized homonegativity and emotion dysregulation in SSIPV perpetration, among a sample of 120 people involved in a same-sex relationship. Our first hypothesis (H1) was not confirmed and no significant direct association between internalized homonegativity and isolating behavior perpetration was found. This might be due to several reasons. First, the lack of significance at a direct level could be motivated by sampling bias. On average, participants reported a high educational level and a high socioeconomic status. Together with low levels of internalized homonegativity, this may have accounted for the non-significance of the data. Second, it is also possible that, as observed in the mediation model, emotion dysregulation nullified the effect of internalized homonegativity, which was, however, not found also at the bivariate level. Third, according to existing literature, internalized homonegativity was generally found to be associated with IPV [19]. However, when considering specific types of violence, results have shown to be more inconsistent. In line with these considerations, it is worth noting that few, if any, data regarding the association between internalized homonegativity and controlling behaviors of isolation exist in the literature, and further studies are needed to deepen these preliminary findings.

A direct and positive association between internalized homonegativity and difficulties in emotion regulation was observed in accordance with H2. The result is in line with current literature [33,35,70,71] and supports the observation that the experience of minority stress, and specifically of internalized homonegativity, may limit one’s access to emotional regulation strategies (such as emotions awareness, emotions acceptance, and cognitive reappraisal). These data are in line with Hatzenbuehler′s Psychological Mediation Framework [32]. One possible explanation for these results may be found within negative affect and attitudes directed toward the self. The adoption of society-negative beliefs and preconceptions may elicit strong emotional responses, which can make it more difficult for people experiencing minority stress to access effective emotion regulation strategies [72]. Consistently, internalized homonegativity has been theorized by Meyer [20] to be a proximal minority stressor involving individual perceptions, appraisals, and emotions. In addition, a greater tendency to use ineffective regulation strategies has been reported in sexual minorities, especially on those days when they encounter minority-stress-related adversities [73]. Further understanding of the relationship between internalized homonegativity and emotional dysregulation may also come from neurophysiological studies. The effect of minority stress on emotion dysregulation could occur through the impact that stress has on the GABAergic system, with an inhibitory effect on the prefrontal cortex, which, in turn, alters the limbic system functions that are in charge of emotion control [74]. The prefrontal cortex is an area that has attracted research interest in relation to minority stress, and there is some agreement that this area is involved in emotion regulation and stress during social rejection [75,76]. 

Furthermore, a direct and positive association between emotion dysregulation and controlling behaviors of isolation was found in accordance with H3, and the mediating role of emotion dysregulation in the relation between internalized homonegativity and isolating controlling behaviors was confirmed as well (H4). These findings confirm previous literature that found emotion dysregulation to be associated with IPV [77,78,79,80], supporting this relation in regard to isolating behaviors as well. In addition, our data support the application of the PMF [32] to SSIPV perpetration. According to the PMF, people with higher levels of internalized homonegativity have shown higher emotion dysregulation, which, in turn, increased the risk of isolating behaviors’ perpetration. A mutual influence between couple relationship dynamics and individual emotion regulation has been documented by several scholars [81,82,83]. Violent behaviors may be seen as a way of avoiding taking contact with uncomfortable emotions regarding one’s intimate relationship and as a distance-regulating mechanism [84,85]. In this sense, isolating behaviors can be seen as a dysfunctional strategy used to control the degree of closeness within the couple′s relationship and thus regulate negative affect that can result from an imbalance between closeness and separation, to which people with low levels of emotional dysregulation can be more vulnerable.

## 5. Limitations and Future Directions

Alongside the results emerging from this study, there are also some limitations, such as the low sample size and a higher percentage of men, which limits the generalizability of our findings. For example, our findings revealed no significant differences between female and male participants in isolating behaviors perpetration. To investigate this issue more reliably, larger samples are needed.

Furthermore, since this is a cross-sectional study, we cannot draw firm conclusions on the actual causal relationships between the variables included in the model and longitudinal data are needed to confirm these preliminary findings. 

Our study was only focused on isolating behaviors’ perpetration, future research should also consider other forms of IPV (e.g., physical, psychological, and sexual violence) and controlling violence (e.g., economic control, threatening control), considering them distinctly rather than as a single phenomenon, as preliminarily introduced in the present work.

Moreover, our study only included gay and lesbian people, while other sexual identities were not included. Bisexual people, for example, have been reported to be the population at greater risk of engaging in IPV by several authors [86,87,88]. Future studies on bisexual people, as well as other sexual and gender minorities, are needed.

Finally, the tested model only explained a low percentage of the variance for isolating controlling behavior (R^2^: 0.64). To better understand predictors of SSIPV and controlling behaviors, future studies should also include other minority stressors (i.e., experiences of discrimination, perceived stigma, and outness) as well as the role of psychological processes and personality dimensions (e.g., adult attachment, identity development, and rejection sensitivity).

## 6. Conclusions

Intimate partner violence is a social concern involving people from different social backgrounds, ethnicities, genders, and sexual orientations. Despite similarities in heterosexual and same-sex couples’ IPV prevalence, SSIPV often remains poorly discussed and represented at the social level. While the literature is growing, there is still a lack of understanding of the complex pathways linking sexual minorities and minority stress to IPV. Our study was one of the first attempts to fill this gap. The emerging results support the extension of PMF to the SSIPV context, although future studies are needed to confirm these data and better understand predictors and mechanisms of IPV perpetration in same-sex couples. The gathered data can also provide useful information at the clinical level and inform services addressing IPV, which nowadays seem poorly sensitive to LGBT+-related issues [15,89]. When dealing with SSIPV, clinicians should avoid erroneously reinforcing the attribution of internal blame and feelings of shame entailed by internalized homonegativity [90]. Instead, while helping sexual minorities facing the impact of minority stress and IPV, mediating psychological variables such as emotion dysregulation may be specifically targeted [90,91]. Treatment and therapeutic process may help identify, learn, and practice coping capacities by increasing emotional regulation strategies, such as cognitive reappraisal, and one’s ability to effectively regulate IPV-involved emotions such as anger [90,92,93]. Considering the indirect impact of internalized homonegativity on SSIPV perpetration, prevention programs aimed at reducing sexual discrimination and social homonegativity are needed to promote both individual and relational well-being in sexual minorities.

## Figures and Tables

**Table 1:** Characteristics of the sample.

	*N*	%
**Sex**		
Female	46	38.3
Male	74	61.7
**Gender ^a^**		
Woman	45	37.5
Man	71	59.2
Transgender	2	1.7
Other	2	1.7
**Sexual Orientation**		
Lesbian	47	39.2
Gay	73	60.8
**Relationship Duration**		
Less than 1 year	16	13.3
1–5 years	57	47.5
6–10 years	27	22.5
11–15 years	7	5.8
More than 15 years	8	6.7
**Educational level**		
Middle school diploma or less	4	3.3
High school diploma	41	34.2
Bachelor’s degree	39	32.5
Master’s degree or higher	36	30
**Employment status ^b^**		
Unemployed	3	2.5
Freelancer	20	16.7
Employee	49	40.8
Student	43	35.8
Homemaker	2	1.7
Retired	3	2.5
**Economic satisfaction**		
Unstable	13	10.8
Sufficient	65	54.2
Wealthy or higher	42	35

Note: *N* = 120. ^a^ 2 missing values. ^b^ 5 missing value.

**Table 2:** Frequencies and mean scores of scales for study variables.

Isolating Behaviors Prevalence	*N*	%
Perpetrators	78	65
Non-perpetrators	42	35
**Study Variables**	***Mean***	***SD***
Isolating behaviors perpetration	0.37	0.42
Internalized homonegativity	1.50	0.57
Emotion dysregulation	2.18	0.58

Note: *N* = 120.

**Table 3:** Bivariate correlations between scale study variables.

	1	2	3
1. Internalized homonegativity	—		
2. Emotion dysregulation	0.46 **	—	
3. Isolating behaviors	0.16	0.22 *	—

Note: *N* = 120. ** *p* < 0.01; * *p* < 0.05.

## Data Availability

The data that support the findings of this study are available on request from the corresponding author.
